# Development of UV-Irradiated PADC and Improvement of Etching for Reducing Experimental Time

**DOI:** 10.3390/ma16155413

**Published:** 2023-08-02

**Authors:** Ippei Ishikawa, Atsushi Kimoto, Shuji Kiyohara

**Affiliations:** National Institute of Technology (KOSEN), Maizuru College, Kyoto 625-8511, Japan; a0474@g.maizuru-ct.ac.jp (A.K.); kiyohara@maizuru-ct.ac.jp (S.K.)

**Keywords:** poly-allyl diglycol carbonate, homemade PADC, radiation education, fading

## Abstract

PADC is well known as a highly sensitive solid-state nuclear track detector. A proposal is for a radiation education method that utilizes these characteristics. A significant issue in the proposed educational method using PADC is the time-consuming etching process. This study attempted to reduce etching time by using a homemade PADC. The experimental results have revealed that the homemade PADC achieves faster etch pit enlargement compared to BARYOTRAK (commercial PADC). An attempt was made to enlarge etch pit diameters rapidly by irradiating UV at a wavelength of 253.7 nm and etching with NaOHaq/ethanol solution. The results revealed that UV irradiation at a wavelength of 253.7 nm, after etching, resulted in etch pit diameters several times larger than those obtained in conventional methods within the same etching time. Therefore, UV irradiation and short-time etching with NaOHaq/ethanol solution proved to be effective. This study also investigated the effects of fading on the PADC during its storage period after UV irradiation. The experimental results confirmed that the etch **pit** diameters shrank by approximately 30% after 2 months of storage. However, considering the enlargement effect of the etch pit diameters due to UV irradiation, it can be concluded that UV irradiation is practical for radiation education experiments.

## 1. Introduction

PADC (Poly-allyl Diglycol Carbonate), well known as CR-39, is a widely used solid-state nuclear track detector (SSNTD) [1]. The CR-39 detector is widely used in various scientific fields, such as nuclear physics, radon dosimetry, and radiobiological experiments. Moreover, PADC track detectors have been suggested as effective and convenient for measuring heavy ion beams [2].

When PADC is irradiated, chemical bonds are cleaved along the radiation track, and the latent track is formed. It is extremely small, ranging over a few nanometers. However, when etching with chemical agents, they preferentially affect the region along the latent tracks, and tracks are enlarged. By etching with alkaline chemical agents, latent tracks can be enlarged to the extent visible under an optical microscope. A method for radiation education using PADC track detectors has been proposed [3]. Radiation is not visible to the naked eye, making it difficult for beginners to understand and visualize through lectures alone. Therefore, it is necessary to enhance understanding through the visualization of radiation using experimental methods. Common radiation detectors such as GM counters and scintillation counters visualize radiation using numerical values. However, for beginners, visualization through numerical values alone may be challenging and may not lead to a concrete understanding. In radiation education in Japan, cloud chambers are currently used as experimental devices. Cloud chambers visualize the tracks of charged particles using the condensation effect of vapor, which is suitable for observing the presence of radiation for beginners. However, cloud chambers have drawbacks such as the need for pre-preparation with dry ice and the instability of visualization capability. Therefore, the objective is to achieve a simplified and more effective understanding of radiation by using PADC to observe damage caused by charged particles. It is believed that by observing the physical damage, it is possible to better understand the energetic nature of radiation compared to other visualization devices [4]. Specifically, after providing basic radiation education to beginners, the PADC is irradiated with alpha particles using radiation minerals or similar sources and followed by etching and observing the particle tracks. However, a drawback of using PADC is the time-consuming etching process required for radiation visualization. Etching is a process in which the bulk etching rate increases with the rise in the concentration and temperature of the etching solution [5]. Therefore, it may be possible to shorten the visualization time by setting higher concentrations and temperatures in radiation education experiments. However, it is important to consider the safety precautions and limitations associated with conducting general educational experiments. Extreme variations in concentration and temperature cannot be feasible due to safety concerns. Therefore, in the laboratory, PADC is being developed with a specific focus on reducing etching time. In the previous research, an appropriate amount of polymerization inhibitor was added during the fabrication of PADC, resulting in a successful reduction in etching time.

Numerous studies showed the influence of ultraviolet (UV) irradiation on the reactions of bulk etching and track etching. Jaleh et al. conducted a study on the effect of pre-irradiation with UV light sources [6]. And Yasir Yahya Kassim and Rabee B. Alkhayat investigated the length and diameter of alpha particle tracks in PADC under different UV irradiation conditions and revealed the enlargement of track diameters due to UV irradiation [7]. Additionally, it has been found that the bulk etching rate of PADC is significantly improved in NaOH aqueous and ethanol solutions compared to the NaOH aqueous solution [8,9]. The same etching mechanism is observed when ethanol is added. However, the etching products generated during the etching process have a high solubility in ethanol. Therefore, by adding ethanol, the bulk etching rate of PADC can be enhanced [10]. Additionally, from the experimental results, it has been observed that when irradiating long-term stored UV-exposed PADC with radiation and subjecting it to etching, a reduction in etch pit diameter (fading) occurs. In the context of using PADC as a radiation education material, the occurrence of fading is undesirable.

In this paper, the effects of both ultraviolet (UV) irradiation and etching with an ethanol-added solution on homemade PADC were evaluated. Additionally, the study investigated the impact of fading during the storage period of PADC that underwent UV irradiation.

## 2. Experiment

**Figure 1:**
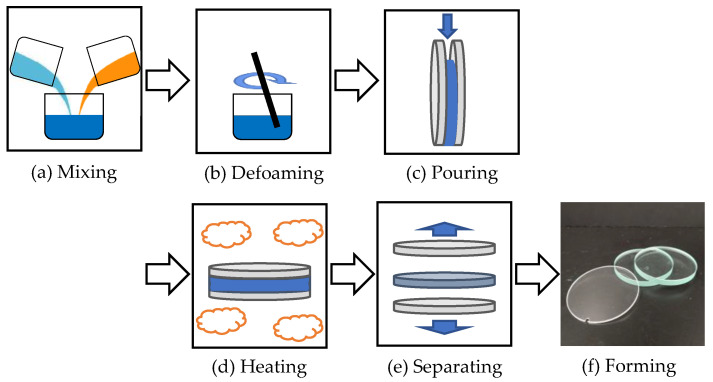
Manufacturing process of homemade PADC.

**Figure 2:**
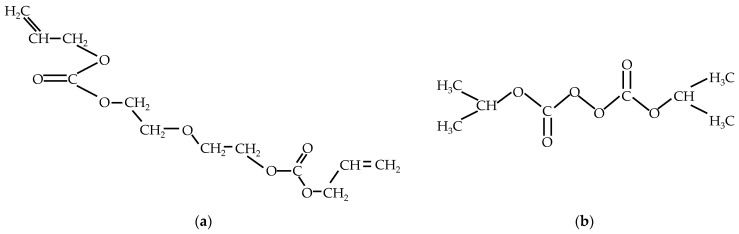
The chemical composition of the mixture: (**a**) ADC (C_12_H_18_O_7_) monomer; (**b**) IPP (C_8_H_14_O_6_) monomer.

**Figure 3:**
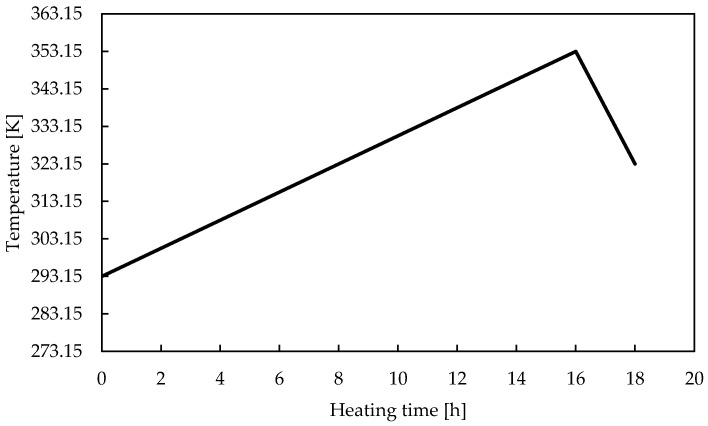
Thermal history during polymerization.

The laboratory produces its homemade PADC as shown in Figure 1 below. PADC is a mixture of ADC (C_12_H_18_O_7_) monomer, as shown in Figure 2a, and IPP (C_8_H_14_O_6_) monomer, as shown in Figure 2b. PADC polymerization is initiated by radicals and proceeds through the addition of allyl groups, forming a dense three-dimensional network of polyaryl chains linked by diethylene glycol carbonate [11]. IPP acts as the polymerization initiator. The two monomers are mixed and deformed using a stirring and defoaming device (THINKY Inc., AR-100, Tokyo, Japan) in a 9:1 ratio, as shown in Figure 1a,b. Subsequently, the mixture is poured into a mold made of soda glass (Tokyo Glassware Co., Ltd., 000-160-30-07, Tokyo, Japan) as shown in Figure 1c. The mold is formed with a thickness of 1 mm. Then, as depicted in Figure 1d and the thermal history shown in Figure 3, thermal curing is performed.

In this study, experiments were conducted to compare the etch pit diameters of homemade PADC using the mentioned method and BARYOTRAK (from Fukuvi Chemical Industry, Ltd., Fukui, Japan) (hereafter referred to as commercial PADC). The homemade PADC was utilized for the specific identification of methyl groups present at the branching points in PADC through FT-IR analysis [12].

The PADC produced using the method described in Figure 1 was cut into a 1 cm square using a diamond cutter (HOZAN Inc., K-111 PCB CUTTER, Japan, Osaka). Subsequently, radiation exposure was performed using a vacuum pump (ULVAC Inc., G-20DA, Kanagawa, Japan) and a collimator, utilizing ^241^Am as the alpha particle standard radiation source. The collimator was employed to generate alpha tracks perpendicular to the surface of the PADC. The ^241^Am source contains ^232^Th and ^238^U and emits alpha particles with an energy of approximately 4–5 MeV. After α-rays irradiation, chemical etching was performed using a 30 wt% sodium hydroxide aqueous solution (referred to as NaOHaq) for 10 min intervals, ranging from 10 to 40 min. The etching process was conducted by stirring at an etching temperature of 90 °C.

**Figure 4:**
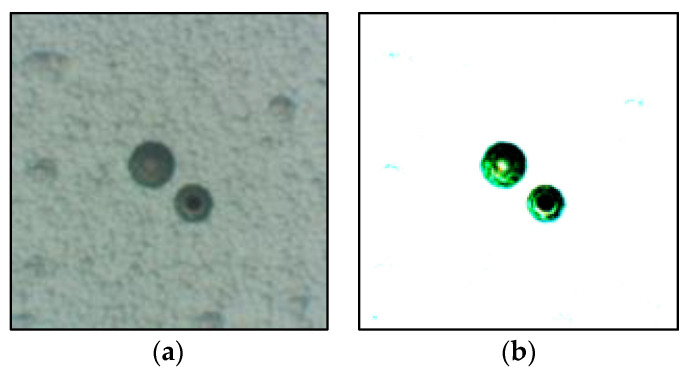
Comparison between the original image and the binary image on a brightness threshold of 90: (**a**) Original image; (**b**) Binary image.

Next, the homemade PADC was irradiated with UV light at a wavelength of 253.7 nm, using a UV irradiation device (DAISHIN KOGYO Inc., DM-90, Osaka, Japan), at a distance of approximately 10 cm. The UV irradiation was conducted at intervals of 1 h within the range of 0 to 15 h. It has been found that ultraviolet radiation with a wavelength of 253.7 nm promotes surface degradation of PADC and increases the bulk and track etching rates [13]. The UV intensity at a distance of 10 cm was 1300 µW/cm^2^. The improved PADC was subjected to chemical etching using a hot magnetic stirrer (IKA Inc., C-MAG HS7 digital, Staufen, Germany) with an etching solution consisting of 30 wt% NaOH aqueous solution with 1 wt% ethanol (hereafter referred to as NaOHaq/ethanol). It has been demonstrated by Tse et al. that performing UV irradiation on PADC after radiation exposure results in a significantly higher track etching rate (*V_t_*) compared to UV irradiation prior to radiation exposure [14]. However, considering the focus on radiation education experiments for beginners, the objective is to observe the tracks immediately after radiation exposure. Therefore, UV irradiation is carried out before radiation exposure, and the UV-irradiated plastic is used for educational objectives. The chemical etching process was conducted by stirring at an etching temperature of 90 °C. Subsequently, observations were made using an optical microscope. The observed images were binarized using a brightness threshold of 90. In Figure 4, a comparison is presented between the binary thresholder images and the original images. The average etch pit diameter was calculated for each sample.

Next, in order to observe the fading of the UV-irradiated PADC, the PADC that underwent 11 h of UV irradiation, and the PADC without UV irradiation were placed in light-protected storage containers and stored for 0, 1, and 2 months. Subsequently, the samples were exposed to alpha particles, and chemical etching was performed using the hot magnetic stirrer. The etching process was carried out for a duration of 10 min. Following that, the etch pits were observed using an optical microscope.

The objective of this study is to reduce the etching time for radiation education experiments. Therefore, it is necessary to observe the etch pit diameter with an inexpensive microscope. In this study, an observable range of etch pit diameters was defined as being more than 10 µm.

## 3. Experimental Results

**Figure 5:**
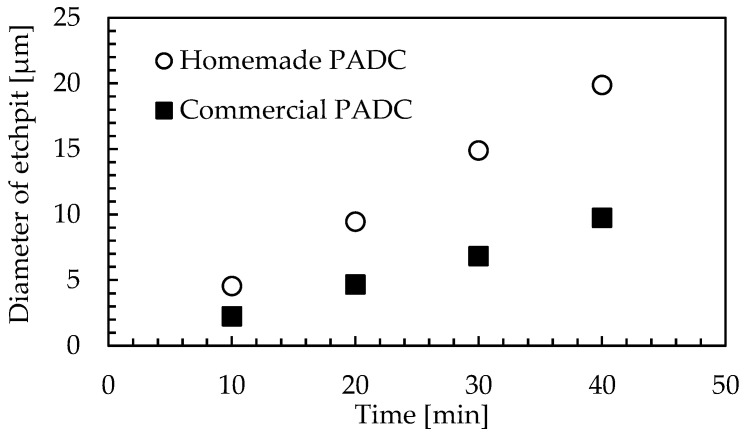
Comparison of etch pit diameters between homemade PADC and commercial PADC.

PADC is dissolved in the etchant by basic hydrolysis of the ester [15]. Figure 5 shows a comparison of etch pit diameters between the homemade PADC and commercial PADC. The etch pit diameters were measured at 15 points on each sample, and the average values were plotted on the graph. Additionally, the etch pit enlargement rate over time was calculated from the plotted etch pit diameters. The results showed that the enlargement rate of the etch pit diameter for the commercial PADC was 0.24 µm/min, while it was 0.49 µm/min for the homemade PADC. The results in Figure 5 show that especially in radiation education experiments, the shortening of the etching process is an issue. With commercial PADC, etching usually takes about 40 min to obtain an etch pit diameter of 10 µm. However, in the case of a homemade PADC, similar results were obtained in about 20 min of etching. The results show the advantage of the homemade PADC.

**Figure 6:**
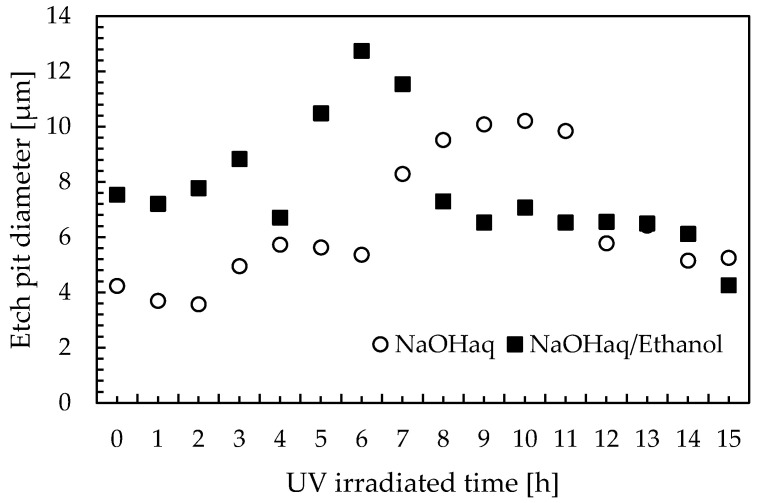
Change in etch pit diameter with UV irradiation time.

**Figure 7:**
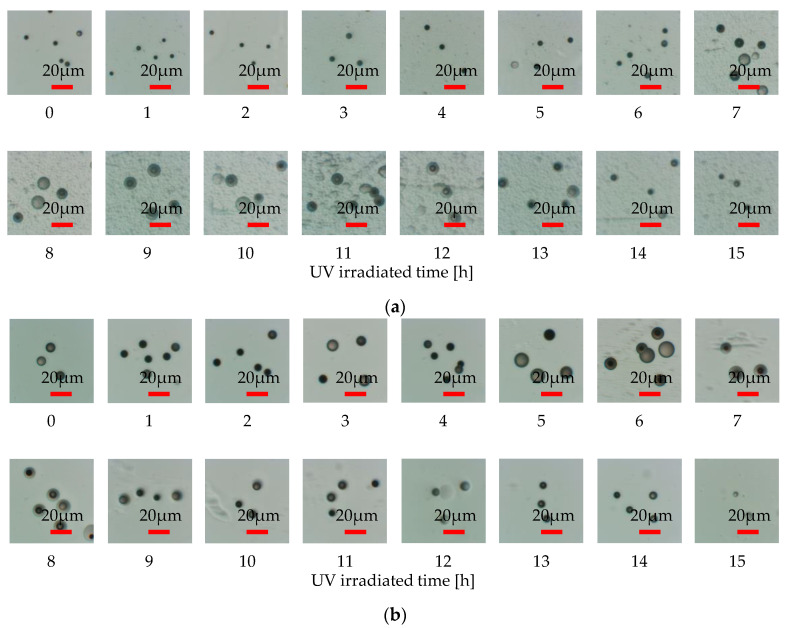
The optical microscope images of the observed etch pits: (**a**) NaOHaq; (**b**) NaOHaq/thanol.

Figure 6 shows the change in etch pit diameters over irradiation time when the homemade PADC is irradiated with UV light. The etching time was fixed at 10 min. The circular markers show the results obtained from etching with NaOHaq, while the square markers show the results obtained from etching with NaOHaq/ethanol. Additionally, Figure 7 shows the observed etch pit diameters. The comparison of observed etch pit diameters at 0 h of UV irradiation reveals that the etch pit diameter for NaOHaq etching is 4.23 µm, while the etch pit diameter for NaOHaq/ethanol etching is 7.53 µm. A comparison of etch pit diameters showed that the addition of ethanol enlarged the pits by a factor of 1.8. This shows that the enhancement of the etching rate with NaOHaq/ethanol is effective even in a short time. As for NaOHaq, the results of observation show that the etch pit diameter was enlarged up to 10.2 µm at 10 h of UV irradiation and then shrank after 10 h. This confirmed that even when UV irradiation was performed on homemade PADC, it had the effect of increasing the bulk etching rate. When comparing the cases where UV irradiation was not performed and the case where it was performed, it was observed that the etch pit diameter increased by approximately twice in terms of diameter ratio. The enlargement of etch pit size by UV is explained according to the Norrish Type I cleavage in the Norrish reaction. This reaction leads to the cleavage of polymer chains, which decreases the average molecular weight of the detector and increases the solubility and diffusion rate of etching products. Therefore, the bulk etching rate is enhanced [16,17]. The formation of hydroxyl or hydroperoxyl groups is also related to the bulk etching rate of PADC. When hydroxyl bonds are established between hydrophilic groups and surrounding water molecules, a structured water layer is formed around the polymer chains [18]. The formation of the water layer explains the excellent solubility of the polymer in sodium hydroxide solution [19]. Similar results were observed with NaOHaq/ethanol. The etch pit diameter was enlarged up to 12.8 µm at 6 h of UV irradiation and then rapidly shrank after 6 h.

**Figure 8:**
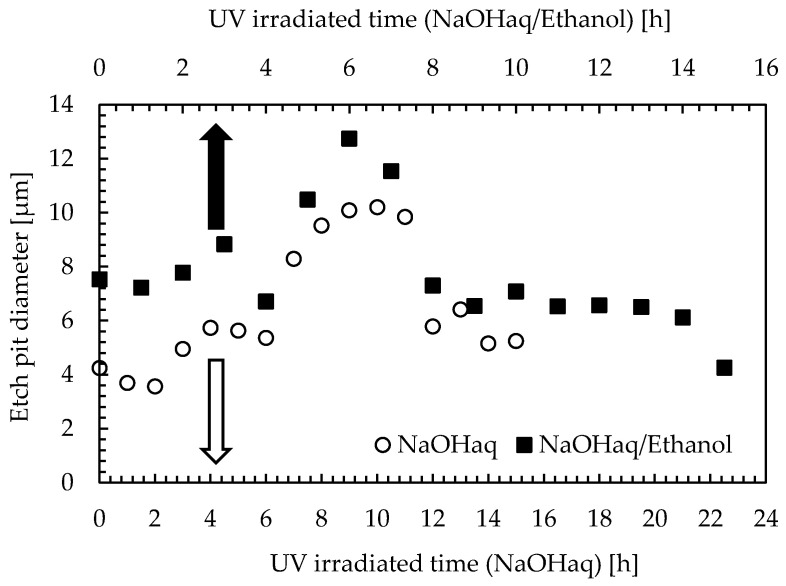
Comparison of the growth behavior of etch pit diameters in NaOHaq and NaOHaq/ethanol. (The growth behavior of NaOHaq and NaOHaq/ethanol was compared by displaying them on both axes).

Figure 8 compares the growth behavior of etch pit diameter in NaOHaq and NaOHaq/ethanol. Similarities were observed when plotting UV irradiation time on both axes for NaOHaq and NaOHaq/ethanol. This result shows that the effect of UV irradiation time and ethanol on etch pit diameter is similar, and the addition of ethanol enhances the UV characteristics.

**Figure 9:**
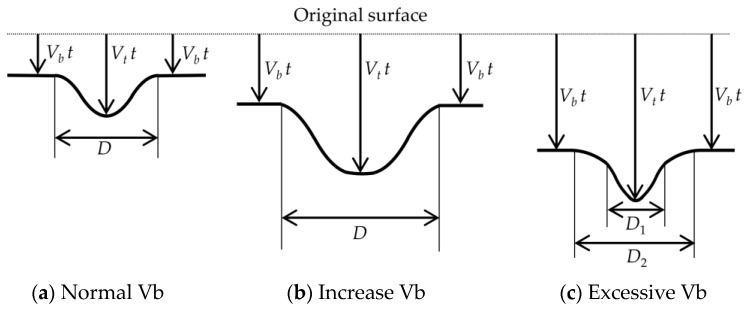
The differences in etch pit formation.

**Figure 10:**
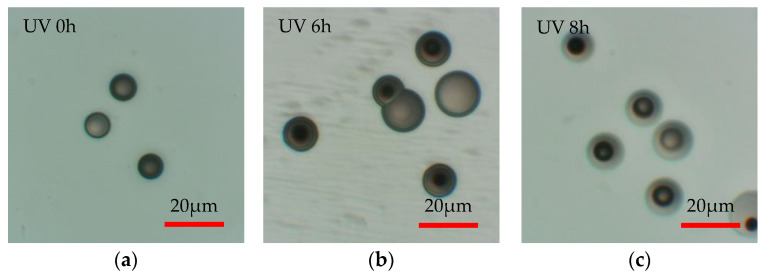
Growth behavior of etch pit diameter under UV irradiation (NaOHaq/ethanol, 30 wt%, 90 °C).

Next, the shrinkage of the etch pit diameter is discussed. When irradiated with UV, the etch pit diameter shrank after 10 h for NaOHaq and after 6 h for NaOHaq/ethanol. This phenomenon is believed to be due to excessive surface activity. Figure 9 illustrates the growth behavior of the etch pit diameter. In normal conditions (a), according to the principle of Huygens, the etch pit diameter *D* is formed into concentric circles [20]. However, when UV is irradiated, the etching rate near the PADC surface increases as shown in (b), leading to increased removal. Therefore, as shown in (c), UV irradiation causes a two-layer etch pit diameter (*D_1_*, *D_2_*) formation by the excessive promotion of *V_b_*. When compared with the etch pit diameters in Figure 7, two-layer etch pit diameters are formed in the range where the etch pit diameter shrinks (NaOHaq: after 10 h; NaOHaq/ethanol: after 6 h). Figure 10 compares NaOHaq/ethanol for UV irradiation times of 0, 6, and 8 h. This result supports the growth behavior of the etch pit diameter shown in Figure 9.

In the experiment, when PADC irradiated UV at a wavelength of 253.7 nm for 6 h was etched with NaOHaq 30 wt%/ethanol 1 wt% (etching temperature: 90 °C) for 10 min, the etch pit diameter was enlarged up to 12.8 µm. As a result, compared to the conventional method (UV 0 h, NaOHaq 30 wt%, 90 °C), it was possible to obtain etch pit diameters up to three times larger with the same etching time. Furthermore, it showed that etch pit diameters of 10 µm or larger, as defined in this study, can be formed within 10 min. One future task is to verify the reproducibility because UV irradiation has experimental variability.

**Figure 11:**
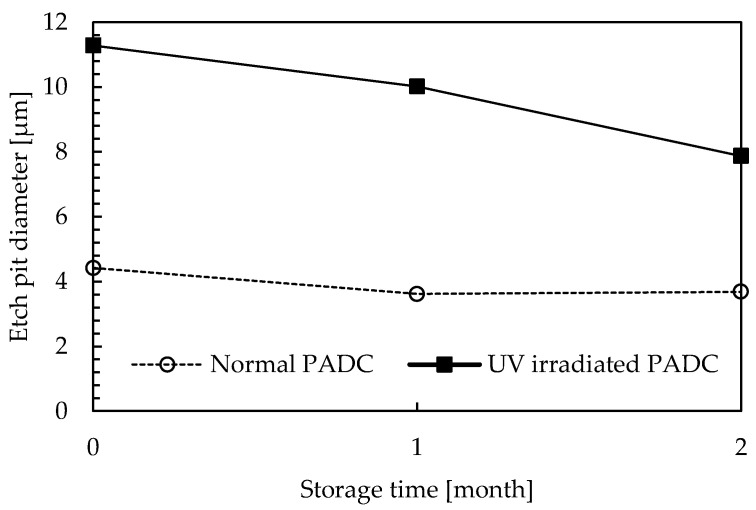
A comparison of the etch pit diameters between PADC subjected to 11 h of UV irradiation and PADC without UV irradiation as a function of storage period.

**Figure 12:**
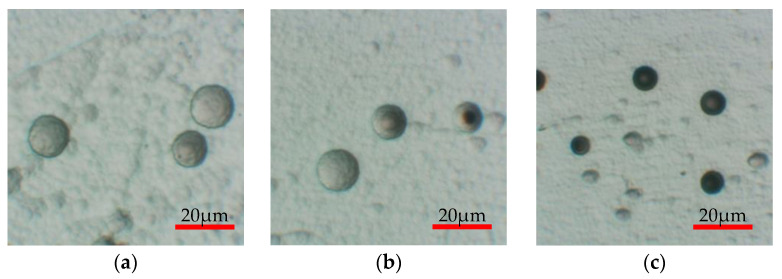
Optical microscope images of etch pits in PADC with different storage periods after 11 h of UV irradiation. (**a**) 0 months; (**b**) 1 month; and (**c**) 2 months.

Next, the effect of fading is discussed. Figure 11 shows a comparison of the etch pit diameters between PADC subjected to 11 h of UV irradiation and PADC without UV irradiation as a function of the storage period. It is shown that the etch pit diameters of the PADC irradiated with UV for 11 h gradually shrank with the storage period. When comparing the 0-month and 2-month storage periods, the etch pit diameter shrank by approximately 30%. On the other hand, the etch pit diameter of the PADC without UV irradiation shows little variation with the storage period, exhibiting a nearly constant diameter regardless of the storage period. Therefore, it is indicated that fading does not occur in the absence of UV irradiation. When using PADC as a radiation education material, the occurrence of fading is undesirable. The cause of fading can be attributed to the production process of stable PADC through the thermal curing of monomers using a polymerization initiator. However, UV-irradiated PADC undergoes Norrish Type I cleavage, leading to the cleavage of polymer chains. This cleavage results in the generation of radicals and the formation of chemically unstable PADC. Radicals can undergo recombination with different radicals, leading to cross-linking. Consequently, it is believed that long-term storage causes the recombination of radicals and the occurrence of fading. Therefore, PADC immediately after UV irradiation and PADC stored for a long period of time are considered to exhibit different compositions. Figure 12 shows the etch pits of PADC subjected to 11 h of UV irradiation for different storage periods. Figure 12a represents the sample where UV irradiation was followed by immediate α-particle irradiation and etching. In this case, spherical defects were observed on the surface of PADC, in addition to the tracks of α-particles. Figure 12b,c represent samples irradiated with UV and stored for 1 month and 2 months, respectively. Compared to Figure 12a, the influence of spherical defects is reduced in Figure 12b,c. This suggests that the radicals in the UV-irradiated region recombine during long-term storage, resulting in fading. However, it has been confirmed that UV irradiation leads to an expansion of the etch pit diameter by more than twice its original size. Even in cases where fading occurs, the reduction in etch pit diameter is only around 30% over a period of 2 months. Therefore, it is considered that UV irradiation still holds practical value for radiation education experiments.

## 4. Conclusions

In this study, with a focus on radiation education experiments for beginners, the objective is to observe the tracks immediately after radiation exposure. A homemade PADC specialized for the visualization of etch pits was fabricated. The effects of both UV irradiation and etching with an ethanol-added solution on homemade PADC were evaluated.

First, the etch pit diameters of homemade PADC were compared with those of commercial PADC. It was observed that the etch pit diameters of homemade PADC increased at a faster rate than those of commercial PADC. Particularly, the homemade PADC showed superiority in the radiation education experiment, which was the objective of this study. Furthermore, when UV irradiation at a wavelength of 253.7 nm was followed by etching, observations revealed significantly larger etch pit diameters, several times larger than the conventional method, within the same etching time. Finally, UV irradiation and short-time etching in NaOHaq/ethanol turned out to be effective approaches.

The effect of UV irradiation on PADC showed that the etch pit diameters gradually shrank with storage time. Fading is caused by UV-induced polymer chain scission, which results in the formation of radicals and chemically unstable PADC. Since radicals combine with different radicals to cause cross-linking, it is thought that long-term storage causes radicals to recombine and cause cross-linking.

## Data Availability

The data presented in this study are available on request from the corresponding author.
